# Systematic review of mitochondrial dysfunction and oxidative stress in aging: A focus on neuromuscular junctions

**DOI:** 10.4103/NRR.NRR-D-24-01338

**Published:** 2025-04-29

**Authors:** Senlin Chai, Ning Zhang, Can Cui, Zhengyuan Bao, Qianjin Wang, Wujian Lin, Ronald Man Yeung Wong, Sheung Wai Law, Rebecca Schönmehl, Christoph Brochhausen, Wing Hoi Cheung

**Affiliations:** 1 Musculoskeletal Research Laboratory, Department of Orthopaedics and Traumatology, The Chinese University of Hong Kong, Hong Kong Special Administrative Region, China; 2 Li Ka Shing Institute of Health Sciences, The Chinese University of Hong Kong, Hong Kong Special Administrative Region, China; 3 Division of Sports Medicine and Adult Reconstructive Surgery, Department of Orthopaedic Surgery, Nanjing Drum Tower Hospital, Affiliated Hospital of Medical School, Nanjing University, Nanjing, Jiangsu Province, China; 4 Institute of Pathology, University Hospital Mannheim, Medical Faculty Mannheim, University of Heidelberg, Heidelberg, Germany

**Keywords:** aging, mitochondrial dysfunction, neuromuscular junction, oxidative stress, sarcopenia, systematic review

## Abstract

Mitochondrial dysfunction and oxidative stress are widely regarded as primary drivers of aging and are associated with several neurodegenerative diseases. The degeneration of motor neurons during aging is a critical pathological factor contributing to the progression of sarcopenia. However, the morphological and functional changes in mitochondria and their interplay in the degeneration of the neuromuscular junction during aging remain poorly understood. A defined systematic search of the PubMed, Web of Science and Embase databases (last accessed on October 30, 2024) was conducted with search terms including ‘mitochondria’, ‘aging’ and ‘NMJ’. Clinical and preclinical studies of mitochondrial dysfunction and neuromuscular junction degeneration during aging. Twenty-seven studies were included in this systematic review. This systematic review provides a summary of morphological, functional and biological changes in neuromuscular junction, mitochondrial morphology, biosynthesis, respiratory chain function, and mitophagy during aging. We focus on the interactions and mechanisms underlying the relationship between mitochondria and neuromuscular junctions during aging. Aging is characterized by significant reductions in mitochondrial fusion/fission cycles, biosynthesis, and mitochondrial quality control, which may lead to neuromuscular junction dysfunction, denervation and poor physical performance. Motor nerve terminals that exhibit redox sensitivity are among the first to exhibit abnormalities, ultimately leading to an early decline in muscle strength through impaired neuromuscular junction transmission function. Parg coactivator 1 alpha is a crucial molecule that regulates mitochondrial biogenesis and modulates various pathways, including the mitochondrial respiratory chain, energy deficiency, oxidative stress, and inflammation. Mitochondrial dysfunction is correlated with neuromuscular junction denervation and acetylcholine receptor fragmentation, resulting in muscle atrophy and a decrease in strength during aging. Physical therapy, pharmacotherapy, and gene therapy can alleviate the structural degeneration and functional deterioration of neuromuscular junction by restoring mitochondrial function. Therefore, mitochondria are considered potential targets for preserving neuromuscular junction morphology and function during aging to treat sarcopenia.

## Introduction

The neuromuscular junction (NMJ) is a chemical synaptic connection between the motor nerve terminal and muscle fibers and is separated by a synaptic cleft (Davis et al., 2022). As the site of transmission for action potentials from motor neurons to muscles, the NMJ enables the propagation of neural signals and subsequent muscle contraction (Rudolf et al., 2014). Age-related NMJ degeneration is a hallmark of sarcopenia and is characterized by a loss of muscle mass and strength (Priyadarsini et al., 2022). Clinically, electrophysiological assessments have consistently shown significant declines in intact motor units across both proximal and distal muscle groups in the upper and lower limbs with aging (de Koning et al., 1988; Stålberg et al., 1989). Furthermore, these studies documented substantial losses of anterior horn cells and ventral root fibers in individuals over 60 years of age (Tomlinson and Irving, 1977). Morphological analyses revealed significant alterations in the NMJ of older adults, including irregularly shaped presynaptic motor nerve endings and a reduced length of the presynaptic membrane. Furthermore, postsynaptic membrane junctional folds degenerate, resulting in a substantial decrease in electron density. These changes suggest that postsynaptic membrane degeneration and consequent focal innervation of the NMJ are the primary events driving age-related endplate changes (Pratt et al., 2021). In animal studies, the NMJ of the extensor digitorum longus (EDL) in aged C57BL/6J mice (29 months old) exhibited an irregular and fragmented morphology, in contrast with the more preserved NMJ in the soleus (Chai et al., 2011). This degeneration is a key contributor to the decline in skeletal muscle mass in older individuals, underscoring the importance of prevention and treatment as crucial research endeavors (Jang and Van Remmen, 2010; Rudolf et al., 2016; Spendiff et al., 2016). Moreover, sarcopenia poses significant challenges for healthcare systems worldwide because of its associated disability, mortality, and high healthcare costs.

Mitochondria are double-membrane organelles that serve as cellular powerhouses and are responsible for generating the majority of cell energy in the form of adenosine triphosphate (ATP) (Gray, 2013). Mitochondria are crucial for maintaining life by generating the majority of the cell’s ATP through oxidative phosphorylation and other energy conversion processes. With advancing age, the integrity and functionality of mitochondrial DNA (mtDNA) decline significantly, resulting from the accumulation of mutations and oxidative damage induced by reactive oxygen species (ROS). The biogenesis of new mitochondria also decreases with age due to alterations in mitochondrial dynamics and the inhibition of mitophagy. The functional decline in mitochondrial oxidative phosphorylation in older individuals has significant implications for exercise capability and muscle strength retention in older individuals (Grevendonk et al., 2021). This decline is accompanied by an increase in ROS and subsequent oxidative stress, which can lead to widespread cellular damage, compromising not only the mitochondrial respiratory chain and calcium homeostasis (Kowalczyk et al., 2021; Chen et al., 2024) but also the pathogenesis of neurodegenerative diseases, such as Alzheimer’s disease, Parkinson’s disease and amyotrophic lateral sclerosis (Kowalczyk et al., 2021). Given their abundance in neurons, muscle fibers, and both sides of the NMJ (Anagnostou and Hepple, 2020), maintaining mitochondrial integrity is essential for preserving neuromuscular health. Moreover, the intricate relationship between mitochondrial dysfunction and neurodegenerative diseases underscores the importance of investigating novel therapeutic strategies to prevent or delay these conditions.

The development of sarcopenia is a complex process initiated by NMJ denervation, which triggers a cascade of events leading to structural and functional decline in the NMJ, ultimately resulting in muscle atrophy. Denervation significantly accelerates this process, contributing to substantial losses in muscle strength and mass (Moreira-Pais et al., 2022). The motoneurons and their terminal endplates, which are deeply embedded within muscles, have emerged as critical targets for sarcopenia intervention. As individuals age, structural and functional alterations in mitochondria in both skeletal muscles and motoneurons become increasingly evident, resulting in oxidative stress and mitochondrial dysfunction, which are major focal points in aging and sarcopenia research (Wiedmer et al., 2021). A key mechanism underlying the involvement of neuronal mitochondria in sarcopenia was elucidated by Rygiel et al. in 2016, highlighting the critical role of mitochondrial dynamics, including swelling, cristae reduction, and impaired fusion/fission balance. This imbalance potentially hinders mitophagy, leading to the accumulation of defective proteins and mutated mtDNA within NMJ terminal boutons. The resulting damaged mitochondria exhibit diminished respiratory chain activity, compromised neuromuscular transmission, reduced membrane potential, and the release of pro-apoptotic factors.

This systematic review provides an in-depth examination of the dynamic changes occurring at the NMJ during aging, including morphological transformations, functional assessments, and alterations in denervation-responsive gene expression levels. We place particular emphasis on the critical roles of mitochondrial biosynthesis, the mitochondrial respiratory chain, and mitophagy in maintaining NMJ integrity throughout life. A comprehensive overview of the animal models employed in the included studies is also provided, highlighting their age characteristics and interventions aimed at mitigating mitochondrial and NMJ degeneration during aging. Furthermore, we summarize the key molecular mechanisms and pathways that regulate mitochondrial biogenesis, the mitochondrial respiratory chain, and mitophagy, offering insights into potential strategies for protecting against mitochondrial and NMJ decline during aging and the progression of sarcopenia. By elucidating these dynamics, this review aims to inform the development of targeted interventions aimed at preserving muscle function and preventing age-related diseases.

## Methods

### Search strategy

A comprehensive literature search was performed across the PubMed, Web of Science and Embase databases (last accessed on October 30, 2024). The search terms included “mitochondria”, “aging” and “neuromuscular junction.” These terms were combined as (mitochondri*) AND (aging OR age*) AND (neuromuscular junction OR NMJ). The same search strategy was applied across all the databases. Adherence to the PRISMA (Preferred Reporting Items for Systematic Reviews and Meta-Analyses) guidelines was ensured, and the study selection process was illustrated using a PRISMA flow diagram.

### Search criteria

The inclusion criteria were as follows: (1) pre-clinical or clinical studies focusing on the role of NMJ in aging; (2) studies investigating the roles of mitochondria and alterations in the aging neuromuscular junction; and (3) full-text articles in English.

The exclusion criteria were as follows: (1) non-English articles; (2) inaccessible full texts; (3) studies not related to neuromuscular aging; (4) studies focusing on distinct diseases; (5) studies without mitochondrial morphology and function assessment within the NMJ; (6) review articles; and (7) conference abstracts, reports or letters.

### Study selection

Two independent reviewers utilized EndNote to manage the results, remove duplicates and screen for relevance. Initial elimination was based on the titles and abstracts, and clearly unrelated studies were discarded. A detailed review adhering to the predefined criteria identified potentially relevant studies. Any discrepancies were resolved through discussion.

### Data extraction

The main data were independently extracted from the included studies by two reviewers (the authors SC and CC) and synthesized in **Tables 1** and **2**, including the authors’ name; year of publication; species and strain; sex; animal model; age range of the animals; interventions; key molecules; major changes in NMJ and mitochondria during aging; and mechanisms regulating mitochondria.

**Table 1:** Characteristics of the included studies

Study	Species, strain	Gender/sex	Animal model	Age	Definition of aging	Sample(s)	Intervention method	Conclusion
Kim et al., 2024	C57BL/6N mice	Male and Female	Normal aging mice	4–14 mon; 18–28 mon	N/A	EDL; GAS; lumbrical muscles	UnAG	UnAG counteracts the loss of muscle mass and strength in old mice. These protections were associated with normalized mitochondrial respiration, ROS generations, and neuromuscular coupling in skeletal muscle of old mice.
Lukasiewicz et al., 2024	Human	Male and Female	N/A	75.9±4.5 yr	N/A	Vastus lateralis muscle	N/A	The abundance of denervation-responsive gene transcripts is a significant determinant of muscle and mobility outcomes in aging humans.
Xu et al., 2024	C57 BL/6J mice	Male	Normal aging mice	16–25 mon	25 mon	EDL; GTN	Nitrone compound OKN-007	OKN-007 treatment ameliorated muscle force loss, reduced NMJ innervation and preserved NMJ morhology in aging mice, but did not protect against muscle mass loss.
Bakooshli et al., 2023	C57BL/6 mice	Male	Normal aging mice	24–26 mon	24 mon	GTN; EDL; SOL	PGDHi, ip	15-PGDH inhibitor restored neuromuscular junctions and function in aged mice.
Tezze et al., 2023	Elegans (Bristol N2 WT and RW1596); C57BL/6JRj mice	Male	Normal aging mice; Opa1–/– mice	22–26 mon	22–26 mon	GTN; EDL; Plantaris	RJx-01 (combination drug of metformin and galantamine)	RJx-01 synergistically improved physical performance and muscle strength in 22-mon-old WT mice and improved skeletal muscle ultrastructure, mitochondrial morphology, autophagy, lysosomal function, and satellite cell content.
Cheon et al., 2023	Drosophila	Male and Female	MFN2/Marf overexpression mice	N/A	N/A	Larvae muscle 6/7 of the A3 segment	Concomitant knockdown of MARK4/PAR-1	Downregulation of MARK4/PAR-1 alliviated the synaptic defects, mitochondrial hyperfusion and respiratory dysfunction caused by MFN2/Marf overexpression.
Thompson et al., 2023	B6; 129P2-*Zmpste24*^tm1Lgf^/J mice	Male and Female	Progeroid Zmpste24–/– mice	5–6 mon	5–6 mon	GTN; SOL	MDSPCs isolated from young mice, ip	Systemic transplantation of young MDSPCs into ZMPSTE24-deficient progeroid mice preserved muscle durance, muscle fiber size, mitochondrial respirometry and NMJ morphology in a sex-specific manner.
Ozes et al., 2023	C57BL/6 mice	Male and Female	Normal aging mice	10 and 18–24 mon	24 mon	Lumbrical muscle, TA, GTN, QUAD and triceps	AAV1.tMCK.NT-3, im	AAV1.NT-3 gene therapy in WT-aged C57BL/6 mice resulted in functional and in vivo muscle physiology improvements, supported by quantitative histology from muscle, peripheral nerves and NMJ.
Merholz et al., 2022	C57BL/6J mice	N/A	Skeletal muscle-specific conditional Csnk2a1–/– mice	2, 4, 6, 8, and 10 mon	N/A	GTN; TA; SOL; Diaphragm	N/A	Conditional knockout of CSNK2A1 resulted in age-dependent muscle weakness accompanied by impaired neuromuscular junction and impaired oxidative metabolism.
Ahn et al., 2022	C57BL/6J mice	Female	Sod1KO/mPRDX3Tg mice	8–10 mon	N/A	GTN; EDL	Muscle-specific overexpression of mPRDX3	Overexpression of PRDX3 in skeletal muscle improved the decrease in muscle quantity and quality caused by excessive mitochondrial hydrogen peroxide production, but not NMJ damage.
Wallace et al., 2021	C57BL/6J mice	N/A	Normal aging mice	16 and 26 mon	26 mon	QUAD; GTN; PLN; SOL; TA; EDL	Ketogenic diet	KD increased NMJ remodeling, mitochondrial biogenesis, oxidative metabolism and antioxidant capacity.
Su et al., 2021	C57BL/6J mice	N/A	i-mn-Sod1^–/–^ mice	2–11, 15–23, and 25–29 mon	25–29 mon	1st lumbrical muscle	N/A	Postnatal Sod1 deletion from motor neurons lead to accelerated aging-related deficits in muscle mitochondrial function and calcium-handling functions, prior to NMJ structural degeneration.
Nichenko et al., 2021	C57BL/6J mice	N/A	Muscle-specific Ulk1 knock-out mice	22 mon	22 mon	TA; EDL; SOL; GTN; Diaphragm	N/A	Ulk1 deficiency resulted in accumulation of ROS producing mitochondria that coincides with reduced muscle force production, denervation.
Graham et al., 2021	C57BL/6 mice; *Drosophila* (42580; 9837; 26650)	N/A	Normal aging mice; third instar larva	Mice: 1, 6, 24 mon	24 mon	Mice forebrain; Larval NMJ	N/A	Overexpression of Rab31 and RhoG and RNAi-mediated knockdown of Mcu promote aberrant NMJ phenotypes.
Hayes et al., 2019	C57BL/6J mice; B6SJL mice	N/A	Sod1^–/–^ mice	4, 12, 20 mon	20 mon	TA; Spinal cord and sciatic nerves	Overexpressing of Miro1	The age-dependent loss of NMJ mitochondria was strongly correlates with NMJ denervation and could be rescued by expression of SOD1. Miro1 improved mitochondrial loss but does not modify denervation.
Ascenzi et al., 2019	FVB mice	Male	Normal aging mice, MLC/IGF-1Ea and MLC/IGF‐1Eb mice	6, 26 mon	26 mon	QUAD; GTN; TA; EDL	Skeletal muscle-specific expression of IGF‐1Ea and IGF‐1Eb	IGF-1Ea or IGF-1Eb improved age-related loss of muscle mass and force, maintain integrity of NMJ, modulate mitochondrial function, ROS detoxification.
Garcia et al., 2018	C57BL/6J mice	Male and Female	Aged mice overexpressing PGC‐1α in skeletal muscle	3, 22, 29–34 mon	22 mon	QUAD; GTN; Heart	Muscle-specific overexpression of PGC-1α	Overexpression of PGC in aging muscle leads to energy metabolism similar to that in young mice, maintaining NMJ integrity and muscle integrity.
Pollock et al., 2017	C57BL/6J mice	Male	B6.Cg-Tg (Thy1-YFP) mice peroneal nerve transection	4–8 mon, 24 mon	24 mon	TA	N/A	Denervated muscle fibers induce mitochondrial peroxide generation in neighboring innervated fibers.
Stemple et al., 2016	Fischer 344xBrown Norway rats	Male	Normal aging rats	6, 30 mon	30 mon	Laryngeal muscle	Neurotrophin-4	NTF4 improved the morphology and functional innervation of aging rat laryngeal muscle.
Sataranataraja et al., 2015	C57BL/6J mice	N/A	Neuro-specific Sod1KO mice	20 mon	20 mon	GTN; SOL; TA; EDL; QUAD	N/A	Neuron specific reduction in CuZnSOD is not sufficient to initiate a full sarcopenia phenotype.
Ivannikov et al., 2015	C57BL/6J mice	N/A	Sod1^–/–^ mice	4–8 mon, 22–28 mon	22–28 mon	LAL	N/A	Decreased muscle strength and NMJ function in SOD–/– mice are associated with ROS-mediated reduction of synaptic vesicle pools and EC uncoupling.
Rygiel et al., 2014	Human	N/A	N/A	Fetus 9–11 wk post-conception; 68–99 yr of age	68–99 yr of age	Lumbar region of spinal cords	N/A	Aging motor neurons showed a decrease in mitochondrial respiratory chain complex I and mtDNA content.
Carnio et al., 2014	C57BL/6J mice; Human	N/A	Muscle-specific Atg7–/– mice	5, 26 mon	26 mon	TA; EDL; GTN; Human muscle biopsies	Overexpression of ATG7; Trolox	Autophagy impairment in muscle induces NMJ degeneration, mitochondrial dysfunction, oxidative stress and profound weakness.
Ibebunjo et al., 2013	Harlan SD rats	N/A	Normal aging rats	6, 12, 18, 21, 24, 27 mon	21, 24, 27 mon	EDL; PLN; GTN; Heart; TA; QUAD	N/A	Reduced mitochondrial function and disruption of NMJ driving rat sarcopenia.
Garcia et al., 2013	Wistar rats	Male	Normal aging rats	4, 24 mon	24 mon	GTN; L4–L6 spinal cord	N/A	Swelling and fusion of mitochondria, Ca2+ overload, increased cytochrome c and caspase 3 at the axon terminal of NMJ.
Jang et al., 2010	C57BL/6J mice	Female	Sod1^–/–^ mice	0–26 mon	18–22 mon	GTN; PLN; SOL	N/A	Increased superoxide in vivo accelerates age-associated muscle atrophy through mitochondrial dysfunction and neuromuscular junction degeneration.
Boaro et al., 2009	Albino Swiss mice	Male and Female	Normal aging mice	3, 6, 9, 13, 17, 21, 25 mon	6, 9, 13, 17, 21, 25 mon	EDL	N/A	Continuous morphological remodeling processes occur throughout the lifetime of the NMJ ultrastructure: mitochondria with alterd creasts in the axon terminal, degenerated junction folders.

COX: Cyto c oxidase; CRC: calcium retention capacity; EDL: extensor digitorum longus; GTN: gastrocnemius; IF: immunoflurescent; im: intramuscular injection; ip: intraperitoneal injection; LAL: Levator auris longus; mtDNA: mitochondrial DNA; OCR: oxygen consumption rate; PTP: permeability transition pore; PLN: plantaris; QUAD: quadricep; SDH: succinodehydrogenase; SOL: soleus; TA: tibialis anterior; TEM: transmission electron microscopy; WB: western blot.

## Results

### Results of the search

**Figure 1:**
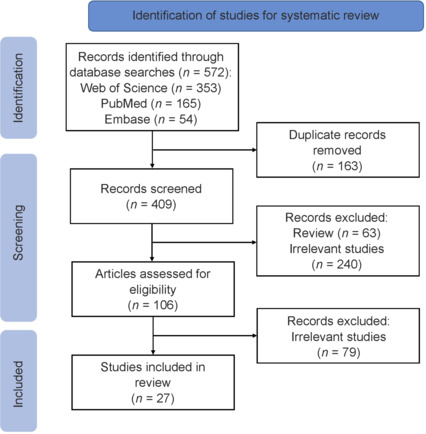
PRISMA (Preferred Reporting Items for Systematic Reviews and Meta-Analyses) flow chart for literature selection.

After the initial search, 353 papers were retrieved from the Web of Science, 165 from the PubMed, and 54 from the Embase databases. In total, 572 papers written in English with full-text availability were selected for further screening. Upon removing 163 duplicate papers, 303 additional papers were excluded based on our selection criteria. A total of 106 papers were identified as potentially relevant for further investigation. Following a more detailed assessment of these publications, 79 irrelevant studies were further excluded. Our results revealed that some excluded studies were primarily conference abstracts or posters without accompanying full-text articles. They are not considered primary sources in our review and would not have altered the overall conclusions drawn from the available literature. As a result, 27 papers were eligible for inclusion in this systematic review (Boaro and Fragoso Neto, 2009; Jang et al., 2010; Garcia et al., 2013, 2018; Ibebunjo et al., 2013; Carnio et al., 2014; Rygiel et al., 2014; Ivannikov and Van Remmen, 2015; Sataranatarajan et al., 2015; Stemple et al., 2016; Pollock et al., 2017; Ascenzi et al., 2019; Hayes et al., 2019; Graham et al., 2021; Nichenko et al., 2021; Su et al., 2021; Wallace et al., 2021; Ahn et al., 2022; Merholz et al., 2022; Bakooshli et al., 2023; Cheon et al., 2023; Ozes et al., 2023; Tezze et al., 2023; Thompson et al., 2023; Kim et al., 2024; Lukasiewicz et al., 2024; Xu et al., 2024). The selection process is presented in **Figure 1**.

### Characteristics of the included studies

The 27 studies included in this review were conducted between 2009 and 2024. The characteristics of the included studies are shown in **Table 1**.

#### Aging models

Normal aging rodent models were used in 12 studies (Boaro and Fragoso Neto, 2009; Garcia et al., 2013; Ibebunjo et al., 2013; Stemple et al., 2016; Ascenzi et al., 2019; Graham et al., 2021; Wallace et al., 2021; Bakooshli et al., 2023; Ozes et al., 2023; Tezze et al., 2023; Kim et al., 2024; Xu et al., 2024). C57BL/6 mice were considered old at 22–28 months of age in 8 studies, and four of them identified 24 months as a critical time point (Graham et al., 2021; Bakooshli et al., 2023; Ozes et al., 2023; Xu et al., 2024). Wallace et al. (2021) used 26-month-old C57BL/6J mice, and Ascenzi et al. (2019) used 26-month-old male FVB wild-type mice as the aged group. Albino Swiss mice aged 3, 6, 9, 17, 21, and 25 months were used by Boaro and Fragoso Neto (2009) for the study, whereas those at 3 months of age were considered young, whereas those at 6 months of age or above were considered old. Stemple et al. (2016) compared 30-month-old and 6-month-old Fisher 344xBrown Norway rats. Garcia et al. (2013) compared 24-month-old and 4-month-old Wistar rats. Ibebunjo et al. (2013) assessed Harlan SD rats that were 6, 12, 18, 21, 24 or 27 months old. Although the old age group was not specifically defined, 24- or 27-month-old rats were selected for comparison with 6- or 18-month-old rats, where their mtDNA content and enzyme and complex activities were significantly decreased.

Oxidative stress and autophagy defects are hallmarks of aging (López-Otín et al., 2023). Some studies have mimicked the pathophysiological characteristics of aged mice: superoxide dismutase 1^–/–^ (*Sod1*^–/–^) mice; autophagy-deficient mice: optic atrophy 1^–/–^ (*Opa1*^–/–^) mice; Unc-51-like autophagy-activating kinase 1^–/–^ (*Ulk1*^–/–^) mice; autophagy-related^–/–^ (*Atg*^–/–^) mice; and gene-edited mice such as zinc metallopeptidase STE24^–/–^ (*Zmpste24*^–/–^) mice and casein kinase 2^–/–^ (*Csnk2a1*^–/–^) mice. ZMPSTE24 is an integral membrane zinc metalloproteinase of the endoplasmic reticulum (ER) involved in the formation of mature lamin A (Bergo et al., 2002). CSNK2 is a serine/threonine-specific protein kinase, and its α-subunit, which has catalytic activity, is responsible for phosphorylating acidic substrates such as casein (Strum et al., 2022). Zmpste24^–/–^ mice at 6 months of age and *Csnk2a1*^–/–^ mice at 10 months of age presented fragmentation and denervation of the NMJ, similar to that of normal aging mice (Merholz et al., 2022; Thompson et al., 2023).

Human samples were used in three studies. Carnio et al. reported significant decreases in the levels of the ATG7 protein and microtube-associated protein 1A/AB light chain 3-II (LC3II) in the muscles of 10 sedentary (sarcopenic) elderly male subjects, indicating a reduction in the autophagy system. ATG7 and LC3II were maintained in the muscles of 4 elderly sportsmen who exercised regularly for long periods of time (Carnio et al., 2014). Rygiel et al. (2014) reported that the protein components of the complex I mitochondrial respiratory chain were reduced or absent in spinal cord samples from 14 older adults (males and females) and that complex I-deficient cells presented reduced mtDNA and a smaller cytosolic size. Lukasiewicz et al. (2024) used transcriptomic data from the vastus lateralis muscles of 575 participants (aged 75.9 ± 4.5 years) and reported that increased expression of denervation responsive genes, including cadherin-15, cholinergic receptor nicotinic alpha 1 subunit, delta submit and epsilon submit), neural cell adhesion molecule 1, RUNX family transcription factor 1 (RUNX1), and sodium voltage-gated channel alpha subunit 5, was negatively associated with exercise capacity, VO_2_ peak, maximal mitochondrial respiration, muscle mass and volume muscle mass and volume, and leg muscle strength (Lukasiewicz et al., 2024).

While laboratory rodents remain the most commonly used animals in aging research, *Drosophila* and *Caenorhabditis elegans* (*C. elegans*) are also being used because of their advantages in terms of cost-effectiveness, accessibility, and ease of genetic manipulation (Piper and Partridge, 2018). Tezze et al. (2023) used RJx-1 to extend the lifespan and improve fitness in *C. elegans*. Graham et al. (2021) promoted abnormal synaptic phenotypes at the NMJ by recapitulating the synaptic mitochondrial proteins Rab31, a member of the RAS oncogene family, Ras homolog family member G and the mitochondrial calcium uniporter in *Drosophila*. Cheon et al. (2023) demonstrated that mitofusin 2 (MFN2)/mitochondrial assembly regulatory factor and microtubule affinity regulating kinase 4/PAR-1 interactions are essential for the maintenance of NMJ synaptic structure and mitochondrial function in *Drosophila*.

#### Interventions to preserve neuromuscular junctions during aging

The interventions for NMJ age-related degeneration reported in the included studies can be divided into four categories: pharmaceuticals, stem cells, a ketogenic diet, and gene editing. The pharmaceuticals included unacylated ghrelin, a peptide hormone consisting of 28 amino acids that does not bind to growth hormone secretagogue receptor 1a. Unacylated ghrelin improves mitochondrial respiration and protects against NMJ disruption in old mice without influencing food consumption, body weight, or adiposity (Kojima et al., 1999). Nitrone compound OKN-007 improved mitochondrial function, protected neuromuscular junction morphology and reduced neuromuscular junction denervation through its potential antioxidant and anti-inflammatory effects in aged mice (Xu et al., 2024). By inhibiting 15-hydroxyprostaglandin dehydrogenase, PGDHi enables the physiological promotion of prostaglandin E2, leading to increased motoneuron viability and the restoration of neuromuscular junctions in aged mice with chronic denervation of muscle (Bakooshli et al., 2023). Tezze et al. (2023) tested the effectiveness of Rjx-01, a combination drug composed of metformin and galantamine, in multiple sarcopenia models. Metformin is widely regarded as the first-line treatment for type 2 diabetes because of its efficacy and safety profile (Halimi et al., 2000). Galantamine has been clinically approved for the treatment of Alzheimer’s disease (Corey-Bloom, 2003). The synergistic effect of the two drugs on Rjx-01 improved autophagy inhibition, mitochondrial morphology and muscle innervation in aged mice. Neurotrophin 4, which is expressed in skeletal muscle, can modulate synaptic efficiency via tyrosine kinase receptors (Stemple et al., 2016). Notably, the intraperitoneal injection of muscle-derived stem/progenitor cells isolated from young female mice into ZMPSTE24-deficient premature aging mice effectively improved muscular endurance, NMJ morphology and mitochondrial function in a sex-specific manner (Thompson et al., 2023). Aged mice fed a long-term ketogenic diet maintained greater muscle mass and better muscle function because of less protein turnover, less protein synthesis, proteasomal degradation and ER stress (Wallace et al., 2021). Overexpression of neurotrophin 3 (Ozes et al., 2023), peroxiredoxin 3 (Ahn et al., 2022), mitochondrial Rho GTPase 1 (Hayes et al., 2019), insulin-like growth factor-1Ea and -1Eb (Ascenzi et al., 2019), PGC-1α (Garcia et al., 2018), and ATG7 (Carnio et al., 2014) or concomitant knockdown of microtubule affinity regulating kinase 4/PAR-1 (Cheon et al., 2023) in mice preserved NMJ during aging.

### Morphological remodeling and denervation dynamics of the aging neuromuscular junctions

A hallmark of NMJ development and maintenance is the continuous process of morphological remodeling, characterized by cycles of denervation and reinnervation. This dynamic process was observed throughout the lifespan. With advancing age, the ability of NMJ to reinnervate becomes compromised. As a result, an increasing number of muscle fibers remain persistently denervated, ultimately contributing to accelerated and progressive muscle dysfunction (Lukasiewicz et al., 2024). Notably, the proportion of denervated NMJs in the fast-twitch muscle group was significantly greater than that in the slow-twitch muscle group (Bakooshli et al., 2023). The disparate vulnerability of fast-twitch and slow-twitch muscle groups to denervation underscores their distinct physiological profiles; however, the underlying mechanisms governing this disparity require further investigation.

Analysis of mRNA expression revealed that markers of skeletal muscle denervation were upregulated with age in both mice and rats (Ibebunjo et al., 2013; Wallace et al., 2021). Denervation-responsive genes are genes that change their expression in response to the loss of nerve supply to muscles. This can occur due to injury, disease, or aging. These genes play crucial roles in the body’s attempt to adapt to and compensate for the loss of neural input. Growth arrest and DNA damage-inducible 45 alpha have been implicated in survival mechanisms, including apoptosis, autophagy, cell cycle arrest and DNA repair (Griñán-Ferré et al., 2024). It responds to environmental stresses by mediating the activation of the p38/Jun N-terminal kinase pathway via protein kinase domain-containing protein/mitogen-activated protein kinase kinase kinase 4 (Takekawa and Saito, 1998). Histone deacetylase 4 (Hdac4) belongs to class II of the histone deacetylase family. HDAC4 expression is markedly elevated in denervated skeletal muscle, and pharmacological inhibition of HDAC4 effectively mitigates denervation-induced muscle wasting (Ma et al., 2021). Myogenin encodes a muscle-specific transcriptional activator involved in the coordination of skeletal muscle development and repair (Vicente-García et al., 2022). Runx1 belongs to the RUNX family of genes, which are also called core binding factor-α. Runx1 plays a critical role in maintaining muscle integrity by preventing degenerative changes in denervated myofibers (Wang et al., 2005). Notably, *Sod1*^–/–^ mice and neuron-specific *Sod1*^–/–^ mice presented NMJ with hallmarks of aging, including increased denervated endplates and increased expression of denervation-responsive genes (Sataranatarajan et al., 2015; Hayes et al., 2019). However, motor neuron-specific *Sod1*^–/–^ mice do not exhibit similar NMJ denervation characteristics (Su et al., 2021). The expression of these denervation-responsive genes was negatively correlated with several key physiological traits in participants, including time to walk 400 meters, VO_2_ peak, maximal mitochondrial respiration, muscle mass and volume, and leg muscle strength (Lukasiewicz et al., 2024). In conclusion, the abundance of denervation-responsive genes is strongly correlated with muscle function in aging humans. This association highlights the importance of developing targeted interventions to restore innervation in advanced age.

### Morphological changes in mitochondria in aging of the neuromuscular junctions

Mitochondrial swelling and the loss of cristae are hallmark signs of mitochondrial injury and dysfunction. Garcia et al. (2013) counted mitochondria in the NMJ of the medial gastrocnemius (left hind limb) of aged rats. The proportion of swollen mitochondria per axon terminal was approximately 66% in old rats but only approximately 9.5% in young rats (4 months). During the preelderly period, some mitochondria within the axon terminal lost cristae, whereas those located beneath the sarcolemma maintained the normal morphology of cristae. With increasing age, the NMJ exhibited a loss and breakage of cristae in the axonal mitochondria, whereas the subsarcolemmal mitochondria swelled. In the advanced stage of aging, the NMJ completely disintegrates, characterized by extensively degenerated mitochondria in the axons, deteriorated junctional folders, and discontinuities in the basal membrane (Boaro and Fragoso Neto, 2009). These findings suggest that mitochondrial degeneration in motor nerve terminals during aging precedes that in the postsynaptic membrane and significantly precedes the degeneration of NMJ structures. The mitochondria in motor neurons could serve as potential targets for preventing NMJ degeneration in early aging.

**Figure 2:**
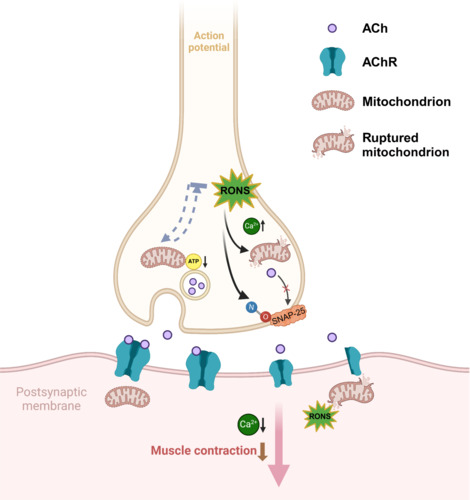
Schematic diagram of the role of mitochondria in neuromuscular junction synaptic transmission. ATP produced by mitochondria mediates the transport of ACh-rich synaptic vesicles and their fusion with presynaptic membranes. Excessive reactive oxygen/nitrogen species cause mitochondria to swell and rupture, releasing mtDNA. Excessive nitric oxide production can induce nitrosative stress during aging and disease. Created with BioRender.com. ACh: Acetylcholine; AChR: acetylcholine receptor; ATP: adenosine triphosphate; RONS: reactive oxygen/nitrogen species; SNAP-25: synaptosome-associated protein-25.

Mitochondrial hyperfusion and the presence of megamitochondria are notable features of mitochondrial morphology in aged cells. In aged rats (24 months), approximately 90% of the axon termini in NMJs presented significant alterations in mitochondria, such as swelling, reduced matrix density, cristae loss, multiple fusions and megamitochondria formation (Garcia et al., 2013). Increased mitochondrial accumulation and increased cristae abnormalities in giant mitochondria in the TA muscle were found in both naturally aging rodents and autophagy-inhibited aging mice (Carnio et al., 2014). Mitochondrial hyperfusion and the formation of megamitochondria imply an imbalance in mitochondrial fusion and fission processes. The genes regulating mitochondrial fission, fission, mitochondrial 1 (*Fis1*), dynamin related protein 1 (*Drp1*), dynamin 1 (*Dnm1*) and fusion (*Mfn1*, *Mfn2*, and *Opa1*), are downregulated with age in the gastrocnemius muscle (GTN) of rats (Ibebunjo et al., 2013). Neurons are highly polarized cells with high energetic demands at their axon terminals, where mitochondria are densely localized. The dynamic distribution of mitochondria in neurons can be modulated by motor proteins and microtubules to facilitate anterograde and retrograde transport (Morsci et al., 2016). The dynamic distribution of mitochondria plays a crucial role in NMJ remodeling and reinnervation during aging. However, giant mitochondria can impede mitochondrial movement along the cytoskeleton. Moreover, mitochondrial fission can facilitate the segregation of damaged mitochondrial segments, thereby promoting their autophagic degradation (Twig et al., 2008). The accumulation of megamitochondria in axon terminals disrupts autophagic clearance, increasing the persistence of damaged mitochondrial components. The molecular mechanisms linking the imbalance in mitochondrial fusion/fission with mitochondrial migration and mitophagy during aging remain understudied (**Figure 2**).

### Functional changes in mitochondria with aging

#### Mitochondrial biogenesis during aging

Mitochondrial biogenesis is a process that depends on two genomes (nuclear genomes and mitochondrial genomes) and is regulated primarily by the transcriptional coactivators (PGC-1 family) of the peroxisome proliferator-activated receptor γ family (Scarpulla et al., 2012). The key molecule that appeared most frequently in the included studies was PGC-1α. Activated PGC-1α leads to increased activation of transcription factor A mitochondria (TFAM), NFE2-like bZIP transcription factor 2 (NRF2) and estrogen-related receptors and promotes the transcription of genes encoding mitochondrial respiratory chain complex subunits. In addition, estrogen-related receptors and NRF2 stimulate TFAM synthesis, which mediates mtDNA replication and transcription (Finck and Kelly, 2006; Ventura-Clapier et al., 2008; Chen et al., 2022). Ibebunjo et al. (2013) reported significantly lower protein levels of PGC-1α in the muscle of aged rats than in those of 6-month-old rats, with statistically significant reductions in mRNA levels even at 12 months of age. Ozes et al. (2023) reported a significant decrease in the relative expression of *Pgc-1α* and mtDNA copy number in the tibialis anterior muscles of 24-month-old C57BL/6 mice. Similarly, Rygiel et al. (2014) reported that reduced mtDNA content in spinal motor neurons in older individuals was associated with a reduction in the protein component of complex I of the mitochondrial respiratory chain. Decreases in the levels of NADH:ubiquinone oxidoreductase subunit B8, NADH:ubiquinone oxidoreductase subunit A9 and ATP5a during aging lead to reductions in the levels of mitochondrial oxidative respiratory chain complexes I and IV (Emelyanova et al., 2018; Kriebel et al., 2020). Decreasing PGC-1α expression levels and activity with aging resulted in reduced mitochondrial biogenesis and decreased oxidative respiratory capacity. Moreover, AMP protein kinase (AMPK) is activated in a low-energy environment and can directly or indirectly phosphorylate PGC-1α, enhancing binding to nuclear receptors and nuclear transcription factors (Jäger et al., 2007). Wallace et al. (2021) reported that the levels of sirtuin 1, sirtuin 3, p-AMPK, AMPK, and phosphorylated p38 map kinase proteins increased with age, whereas PGC-1α levels remained unchanged in C57BL/6J mouse GTN muscles. Ascenzi et al. (2019) reported no significant changes in the relative mRNA expression of *Pgc-1α*, *Nrf2*, or *Mfn2* in the quadriceps muscle of 26-month-old FVB wild-type mice. NAD^+^ insufficiency stimulates SIRT1 nuclear translocation to deacetylate PGC-1α, which activates the expression of genes involved in mitochondrial biogenesis (Tang, 2016; Wu et al., 2022). These are thought to be the body’s self-regulatory mechanisms in response to mitochondrial dysfunction and energy deficiency during early aging.

**Figure 3:**
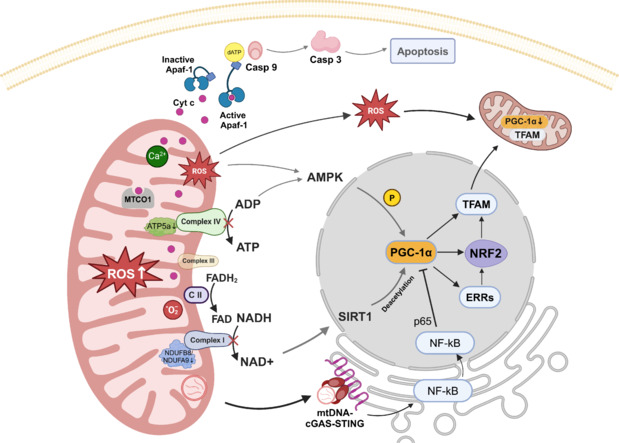
The mechanism of mitochondrial oxidative stress mediates mitochondrial biosynthesis disorders, inflammation and apoptosis during aging. The accumulation of calcium ions in mitochondria leads to increased mitochondrial membrane permeability. Damaged mitochondria release cyt c to activate Apaf-1, which induces apoptosis. Decreases in NDUFB8, NDUFA9 and ATP5a during aging lead to reductions in mitochondrial oxidative respiratory chain complexes I and IV. ATP deficiency activates the AMPK pathway, leading to the phosphorylation of PGC-1α. NAD^+^ insufficiency stimulates SIRT1 nuclear translocation to deacetylate PGC-1α. PGC-1α can increase mitochondrial biosynthesis by promoting the expression of transcription factors such as ERRs, NRF2 and TFAM. mtDNA triggers the activation of the cGAS-STING pathway, leading to increased nuclear translocation of NF-κB and subsequent suppression of PGC-1α by the p65 subunit. Created with BioRender.com. AMPK: AMP-activated protein kinase; Apaf-1: apoptotic protease activating factor 1; ATP: adenosine triphosphate; Casp 9: Caspase 9; cGAS: cyclic GMP-AMP synthase; Cyt c: cytochrome complex; dATP: deoxyadenosine triphosphate; ERRs: estrogen-related receptors; FAD: flavin adenine dinucleotide; NF-κB: nuclear factor-kappa B; NRF-2: NFE2-like bZIP transcription factor 2; PGC-1α: peroxisome proliferator-activated receptor gamma coactivator; ROS: reactive oxygen species; SIRT1: sirtuin 1; STING: stimulator of interferon genes; TFAM: mitochondrial transcription factor A.

An increase in total mitochondrial content was also observed in aging Fischer 344xBrown Norway rat thyroarytenoid muscles via Gomorori’s trichrome staining (Stemple et al., 2016). *Pgc-1α* overexpression in muscle significantly increased mtDNA levels (normalized to the nuclear-encoded gene) but did not increase deletion breakpoint levels (Garcia et al., 2018). In normal aging Harlan SD rats, the content of mtDNA was comparable at 6 and 18 months but decreased significantly at 24 months (Ibebunjo et al., 2013). Additionally, a portion of spinal cord motor neurons in elderly individuals (68–99 years of age) appear to have smaller neuronal cell sizes and reduced mtDNA (Rygiel et al., 2014). A correlation exists between the mtDNA content and PGC-1α expression in the muscles of aged mice. Further research is needed to study this relationship in motor neurons. The decline in mtDNA and PGC-1α levels with aging differs among muscle groups, echoing the distinct responses of fast- and slow-twitch muscles to denervation. mtDNA leaks into the cytoplasm when mitochondrial membrane permeability increases (Luo et al., 2022). mtDNA triggers the activation of the cyclic GMP-AMP synthase-stimulator of interferon genes (cGAS-STING) pathway, leading to increased nuclear translocation of nuclear factor-kappa B and subsequent suppression of PGC-1α by the p65 subunit (Chen et al., 2016). More details are shown in **Figure 3**.

*Csnk2a2*, a subunit of the protein kinase CSNK2, plays a crucial role in cell myogenesis and homeostasis in adult muscle and can interact with the receptor tyrosine kinase MuSK to influence NMJ homeostasis (Hashemolhosseini, 2020). Skeletal muscle from *Csnk2a2*^–/–^ mice exhibited age-dependent impairments in neuromuscular transmission, oxidative metabolism, autophagy, and mtDNA levels (normalized to Pecam1) (Merholz et al., 2022). Ahn et al. (2022) reported increased mitochondrial H_2_O_2_ generation but an insignificant change in mtDNA copy number in the muscle of *Sod1*^–/–^ mice. These findings suggest that the decrease in mtDNA copy number may be related to muscle fiber denervation and altered mitochondrial autophagy rather than mitochondrial oxidative stress.

#### The mitochondrial respiratory chain during aging

As mentioned earlier, a portion of spinal cord motor neurons in elderly individuals appear to have a smaller neuronal cell size and reduced mtDNA, which are associated with a deficiency of complex I but not with cyclooxygenase and succinate dehydrogenase activity (Rygiel et al., 2014). Increased glycogen-positive muscle fibers in the arytenoids of aged rats suggest a reduction in mitochondrial aerobic respiratory capacity (Stemple et al., 2016). Semi-quantification of mitochondrial enzymes and complexes in gastrocnemius muscle homogenates revealed that the activity of citrate synthase in the soluble (S20) fraction and of complexes I, II, and IV in the pellet (P20) fraction decreased gradually with age when standardized to muscle weight but not when normalized to protein mass. This might be caused by a decrease in muscle mass and a decrease in protein production per milligram of muscle with age (Ibebunjo et al., 2013). However, citrate synthase activity, the oxidative phosphorylation protein NADH:ubiquinone oxidoreductase subunit B8 and mitochondrially encoded cytochrome c oxidase I) were significantly greater in the gastrocnemius muscle of 26-month-old mice than in that of 16-month-old mice, as reported by Wallace et al. (2021). Muscle strength was not diminished in middle-aged (15–23-month-old) C57BL/6J mice, but the amount and rate of mitochondrial NADH reduction during contraction were greater than those in young (2–11-month-old) C57BL/6J mice, indicating a decline in NADH utilization efficiency prior to the manifestation of weakness. The decline in mitochondrial function in muscle preceded NMJ degradation and muscle strength decline and was directly regulated by neuronal redox status during aging (Su et al., 2021).

*Sod1*^–/–^ mice exhibit NMJ degeneration and muscle atrophy similar to those observed in age-related sarcopenic mice (Jang et al., 2010; Ivannikov and Van Remmen, 2015; Sataranatarajan et al., 2015; Su et al., 2021; Ahn et al., 2022). Compared with those of young wild-type mice, the frequency of spontaneous neurotransmitter release, the amplitude of evoked neurotransmitter release, the synaptic vesicle pool size and excitation contraction uncoupling were significantly lower in young *Sod1*^–/–^ mice (4‒8 months) and old wild-type mice (22‒28 months). In old mice, both the basal (unstimulated) and stimulated (10 Hz, 30 minutes) rates of ROS production were significantly increased in presynaptic mitochondria within LAL muscles. In stimulated (30/100 Hz, 2 seconds) young *Sod1*^–/–^ and old wild-type mice, presynaptic cytosolic calcium concentrations and mitochondrial calcium uptake amplitudes substantially increased, whereas sarcoplasmic calcium flux was reduced in LAL muscle fibers (Ivannikov and Van Remmen, 2015). Aged *Sod1*^–/–^ mice (18–20 months) presented an increased number of mitochondria around the NMJ but also significant mitochondrial swelling, increased ROS production, decreased ATP production and respiratory control ratio, damage to mitochondrial membrane integrity, denervated NMJ and fragmentation of AChR (Jang et al., 2010). When Sod1 was specifically deleted from neurons, it was not sufficient to trigger a full sarcopenic phenotype. Compared with wild-type mice, n*Sod1*^–/–^ mice (20 months) presented altered NMJ morphology; a slight decrease in the maximum isometric specific force in the GTN and EDL muscles; a slight reduction in quadriceps and soleus muscle mass (but no reduction in GTN, TA or EDL muscle mass); and no increase in mitochondrial ROS production or oxidative stress levels in muscle (Sataranatarajan et al., 2015). In a study by Su et al. (2021), n*Sod1*^–/–^ mouse muscles exhibited reduced force generation, decreased mitochondrial and calcium handling capacity, and stable NMJ morphology at middle age (20 months). Altered neuronal redox status leads to a decline in muscle mitochondrial function before structural changes in NMJ or loss of strength in middle age (Su et al., 2021). In summary, altered neuronal redox status leads to reduced muscle mitochondrial function but is not sufficient to trigger a full muscle loss phenotype.

#### Mitophagy during aging

Autophagy is a process of intracellular homeostasis that involves the destruction and recycling of the cytoplasm, proteins, and organelles by lysosomes (Parzych and Klionsky, 2014). The most studied type of autophagy is macroautophagy, and the degradation of mitochondria by the macroautophagy pathway is known as mitophagy (Zhang, 2013). Mitophagy is essential for removing damaged mitochondria to maintain appropriate mitochondrial quality control (Ding and Yin, 2012; Li et al., 2021). Autophagy levels were significantly reduced in both aged mice and elderly muscles, as evidenced by decreased protein levels of ATG7 and LC3II/LC3I. The inhibition of autophagy in mice by the knockout of *Atg7* resulted in the accumulation of abnormal mitochondrial morphology, decreased mitochondrial membrane potential, impaired mitochondrial function, enhanced oxidative stress, and degeneration of the postsynaptic membrane and AChR in muscles (Carnio et al., 2014). Nichenko et al. (2021) reported that the level of phosphorylated ULK1, an autophagy-related kinase that initiates autophagosome assembly and participates in autophagosome degradation, was reduced in aged mice (22 months) and elderly (60–80 years) muscles. The basal and fasting autophagy flux results suggested that muscle-specific *Ulk1*^–/–^ mice had an impaired ability to simulate autophagy flux. In muscle-specific *Ulk1*^–/–^ mice, State III (malate/glutamate/succinate/saturated adenosine diphosphate) and uncoupled (FCCP-uncoupled) respiration are reduced, and the ROS per unit of oxygen flux is increased in permeabilized muscle fibers (Nichenko et al., 2021). When PGC-1α is overexpressed in the skeletal muscle of aged mice, age-related changes in the NMJ are minimized, as mentioned above, and the levels of the autophagy markers BECLIN1, LC3II/LC3I, WPP domain interacting protein 2, and phosphorylated WPP domain interacting protein 2 are markedly increased (Garcia et al., 2018). Caloric restriction and exercise can improve aging-induced decreases in mitochondrial function and oxidative stress by enhancing mitophagy, thereby improving muscle strength and maintaining NMJ stability (Lanza et al., 2012; Carnio et al., 2014; Joseph et al., 2016; **Table 2**).

**Table 2:** Major changes in muscle function, NMJ, and mitochondria during aging

Study	NMJ	Mitochondria	Key molecule	Mechanism	Connection between NMJ and mitochondria
Kim et al., 2024	UnAG normalized sciatic nerve-stimulated force in GAS and inervated NMJ in aged mice.	UnAG improves mitochondrial respiration and ROS generation rates in skeletal muscle of old mice	UnAG	OXPHOS; ROS production	UnAG decreased mitochondrial respiration, increased hydrogen peroxide generation and reversed NMJ impairment in skeletal muscle of old mice.
Lukasiewicz et al., 2024	CDH15, CHRNA1, CHRND, CHRNE, NCAM1 were negatively associated with SOMMA participant traits.	Maximal mitochondrial respiration was negatice associate with denervation-responsive gene	NEFM, CDH15, RUNX1, CHRNA1, CHRND, NCAM1, MUSK, CDK5R1, CAV3, SCN4A, and SCN5A	OXPHOS	Maximal mitochondrial respiration have strong negative associations with 19 denervation-responsive genes.
Xu et al., 2024	Morphology: a reduced loss of innervation at NMJ and well-preserved NMJ morphorlogy with OKN-007 treatment.	Function: OCR of complex I+II increased with OKN-007 treatment	OKN-007	Antioxidant	OKN-007 improved mitochondrial function through antioxidant properties and reduced NMJ denervation in aged mice.
Bakooshli et al., 2023	Morphology: Axonal swelling, NMJ fragmentaion and denervation.	VDAC1, PDHA1 ↑ in DN Myo	15-PGDH	Possitive corr. with 15-PGDH: FoxO signal, TGF-β signal, Autophagy; Negative corr. with 15-PGDH: OXPHOS, TCA cycle, Glycolysis/gluconeogen, AMPK signaling	Low VDAC1, PDHA1 and high LC3A, Parkin in denervated Myo.
Tezze et al., 2023	Morphology: NMJ denervation was rescued by RJx-01 treatment.	Morphology: Damaged and swollen mitochondria	OPA1	OXPHOS; Mitophagy	Opa–/– mice showed more denervated NMJ and poor muscle function, rescued by Rjx-01.
Cheon et al., 2023	Morphology: synaptic bouton loss	Morphology: Mitochondrial hyperfusion; Function: Respiratory dysfunction induced by MFN2/marf	MFN2/Marf; MARK4/PAR-1	Mitochondrial dynamics, OXPHOS	Overexpression of MFN2/Marf decreased OXPHOS activity and the number of synaptic boutons, MARK4/PAR-1 knockdown rescued it.
Thompson et al., 2023	Morphology: AChRs number, length, and area ↓	Function: Mitochondrial respiration rate and Critrate synthase rate ↓	ZMPST24	OXPHOS	Young MDSPCs transplantation preserved mitochondrial respirometry and NMJ morphometrics.
Ozes et al., 2023	Morphology: myelin thickness ↓; denervation	Biogenesis: Pgc1α ↓, Cox1↓, Cox3 ↓, Atp5d ↑; mtDNA ↓	NT-3	mTORC1; OXPHOS	NT-3 increased mtDNA copy number and NMJ innervated.
Merholz et al., 2022	Morphology: Heavily fragmented NMJs; molecules: synaptic gene expression levels ↑; electrophysiology: EPP, mEPP amplitude, mEPC amplitude, mEPC frequency ↓	Function: cytochrome oxidase ↑ and mitochondrial enzyme activity underneath sarcolemma accumulation; Biogenesis: mtDNA relative quantitation ↓	CSNK2A1	OXPHOS; autophagy	Knockout of CSNK2A1 resulted in age-dependent NMJ damage and impaired oxidative metabolism.
Ahn et al., 2022	Morphology: NMJ area and fragmentation ↑; function: nerve stimulated force deficit; molecules: AchR5-α, GADD45-α, Runx1, sarcolipin mRNA levels ↑	Function: Rate of hydrogen peroxide generation ↑; OCR ↓; CRC ↓	SOD1	H2O2 production	NMJ disruption with H2O2 overproduced; Muscle-specific PRDX3 overexpression reduces mitochondrial H2O2 generation, improves mitochondrial function, despite persisting NMJ impairment.
Wallace et al., 2021	Molecules: mRNA expressions of denervation marker GADD45-α, Hdac4, Runx1 and AchR subunits: Chrnd, Chrng ↑	Biogenesis: SIRT1, SIRT3, AMPK, p-p38MAPK ↑; OXPHOS: NDUFB8, UQCRC2, MTCO1 ↑; SOD2 ↓; Autophagy: LC3 ratio ↓, FOXO3A ↑	PGC-1α	AMPK, p38MAPK, mTOR pathway	Ketogenix diet increased markers of NMJ remodeling, mitochondrial biogenesis, oxidative metabolism and antioxidant capacity.
Su et al., 2021	Morphology: No obvious denervation in i-mn-Sod1^–/–^ mice	The dynamics of NADH response to contractions is impaired in middle age	SOD1	Antioxidant	Specific deletion of neuronal Sod1 inducted an acceleration of age-associated deficits in muscle mitchondria and calcium handling function prior to NMJ deterioration.
Nichenko et al., 2021	Morphology: More innervated fibers and less denervated fibers in aged Ulk1 knockout mice	Biogenesis: mtDNA/nuclear DNA ↓; OXPHOS: OCR, citrate synthase activity, H2O2 production ↑ in aged Ulk1 knockout mice	ULK1	Autophagy	Age related Ulk1 deficiency contributed to decreased autophagy flux and accumulation of dysfunctional mitochondria.
Graham et al., 2021	Morphology: Rab31 and RhoG overexpression and Mcu knockdown decreased total bouton area, active zone punctate area	451 differentially expressed mitochondrial candidates associated with synaptic stability during aging	Rab31, RhoG, Mcu	Methylation; DNA-binding; transit peptide; lipoprotein; symport	96 mitochondrial-associated candidates may have the propensity to modulate synaptic stablity during aging.
Hayes et al., 2019	Morphology: Denervation endplate increase in Sod1^–/–^ mice	A trend toward increasing mitochondrial loss	SOD1; Miro1	Antioxidant; mitochondrial transport	Loss of mitochondria in the diatal motor axon contribute to NMJ denervation via calcium buffering or energy production.
Ascenzi et al., 2019	Morphology: Integrity of NMJ was mainteined preserved in MLC/IGF-1Ea and MLC/IGD-1Eb mice during aging	Aged IGF-1Ea mice: increased ROS production and markers ofanti-oxidant activity (PGC1-α and Nrf-2); increased mRNA of MFN2 and MTFP1	IGF-1	AMPK; autophagy; OXPHOS	The local expression of IGF-1Ea or IGF-1Eb can modulate mitochondrial function, ROS detoxification and preserved NMJ morphological integrity.
Garcia et al., 2018	Morphology: NMJ segmentation; Molecules: Asymmetric A8 and A12 AChE forms ↓	PGC-1α overexpression mice: mitochondrial biogenesis increased: SDHA, ATP5A, UQCRC2, NDUFA9, COX IV, LC3II/LC3I, BECLIN 1, p-WIP2 ↑; age-related mtDNA deletion levels no change	PGC-1α	Autophagy; OXPHOS	PGC-1α overexpression increased mitochondrial biogenesis and minimized age-related changes in the NMJ.
Pollock et al., 2017	Peroneal nerve transection mice: lack of presynatic input, no significant morphological changes in postsynaptic structures within 10 days	Mitochondrial generation of H2O2 increased in denervated muscle	Monoamine oxidase B; NADPH	OXPHOS	Recently, denervated fibers leads to increased ROS generation by mitochondria in neighboring innervated fibers.
Stemple et al., 2016	NTF4 treatment: NMJ number ↑	More mitochondria clusters in untreated aging muscle	NTF4	Trk	NTF4 changed fiber size, glycolytic capacity, mitochondrial, tyrosine kinase receptors (Trk), NMJ content, and denervation in aging rat thyroarytenoid muscles.
Sataranatarajan et al., 2015	Morpholoagy: NMJ endplate area ↓, no fragmentaion; denervation: mRNA expressions of AChRα, Runx1 and GADD45α ↑	ROS generation and oxidative stress were not increased in muscle from the nSod1KO mice	CuZnSOD	Antioxidant	Neuronal loss of CuZnSOD initiated alterations at NMJ, but are not sufficient to initiate a full sarcopenic phenotype.
Ivannikov et al., 2015	Function: EMG: EJP amplitude, mEJP amplitude ↓; EYFP fluorescence in LAL nerve terminals changed	Mitochondrial calcium uptake ↑	SOD1	Antioxidant	Young Sod1^–/–^ mice showed NMJ changes seen in old mice, might in part be driven by ROS mediated EC uncoupling.
Rygiel et al., 2014	Morphology: Area of motor neuron cell bodies ↓	Biogenesis: mtDNA content ↓; OXPHOS: Protenis of Complex I ↓	cll-70, cl-19	OXPHOS	Mitochondrial dysfunction in complex I reduced motor neurons could lead to the cell loss and ultimately denervation of muscle fibers.
Carnio et al., 2014	Morphology: NMJ fragmentaion ↑	Morphology: Accumulation of abnormal mitochondria displaying alterations in size, cristae morphology and matrix density	Atg7	Autophagy	Age-related deterioration of synaptic structure and
	moleculer: NCAM, AChRγ, MuSK↑ with Atg7 deficency	Function: Mitochondrial membrane potential ↓			function is exacerbated by defective autophagy.
Ibebunjo et al., 2013	Moleculer: mRNA of NMJ remodeling ↑	Biogenesis: mtDNA↓; mitochondrial fusion and fission, energy metabolism (Citrate synthase, Complex I, II, IV) ↓	PGC-1α	ERRα; Fabp3; Eef1b2; TFAM; TAC;	The depletion of mitochondrial energy metabolism proteins and NMJ proteins during aging were most significantly correlated with sarcopenia.
Garcia et al., 2013	Moleculer: Cytochrome c and activated caspase 3 in the cytoplasm of axon terminals at NMJ	Morphology: Swelling, mitochondrial fusion and megamitochondria in the axon terminals	Caspase 3	Ca2+ overload; Apoptosis	Dramatic alterations of mitochondria and colocalization of dynein and cleaved caspase 3 in axon terminals.
Jang et al., 2010	Morphology: AChRs fragmentation ↑; moleculer: AChRα mRNA ↑; AChRα preotein ↓ in Sod1^–/–^ mice	Morphology: Mitochondrial area and density ↑; extramitochondrial superoxide; Mitochondrial superoxide, H2O2 generation ↑; ATP production; RCR ↓; mitochondrial mediated apoptosis ↑: Bcl-2, Bax, Bak, Cytochrome C, AIF	SOD1	Antioxidant; OXPHOS; Apoptosis	The superoxide-induced NMJ degeneration and mitochondrial dysfunction are potential mechanisms of sarcopenia.
Boaro et al., 2009	Morphology: Degenerated myelin in the cytoplasm of Schwann cells, pleomorphic, multivesiclar bodies and junction folders	Morphology: Mitochondrial degeneration in axon termials	N/A	N/A	Mitochondrias with morphologically altered crests in the axon terminal and degenerated junction folders were frequent and visible with aging.

AchR: Acetylcholine receptor; AMPK: AMP-activated protein kinase; ATG7: autophagy related 7; Bnip3: BCL2/adenovirus E1B interacting protein 3; CDH15: calciumdependent intercellular adhesion glycoprotein; Chrna: cholinergic receptor nicotinic alpha; CMAP: compound muscle action potential; COX: cyto c oxidase; CRC: calcium retention capacity; DN: denervated; Drp1: dynamin related protein 1; EDL: extensor digitorum longus; EE: energy expenditure; EMG: electromyography; EPP: endplate potential; EPC: endplate current; GADD45-α: growth arrest and DNA damage-inducible protein GADD45 alpha; Gclm: glutamate-cysteine ligase, modifier subunit; GTN: gastrocnemius; Hdac4: histone deacetylase 4; MAP1LC3B: microtubule associated protein 1 light chain 3 beta; MFN2: mitofusion2; mPRDX3: mitochondrial peroxiredoxin3; MIRO1: Miro1, mitochondrial cargo adaptor 1; mtDNA: mitochondrial DNA; MTCO1: mitochondrial cytochrome c oxidase 1; Mtfp1: mitochondrial fission process 1; MUSK: muscle associated receptor tyrosine kinase; Myog: myogenin; NDUFB8: NADH ubiquinone oxidoreductase subunit B8; NCAM: neural cell adhesion molecule; NTF4: neurotrophin 4; OCR: oxygen consumption rate; OPTN: optineurin; OXPHOS: oxidative phosphorylation; PDHA1: pyruvate dehydrogenase; PGC-1α: peroxisome proliferator-activated receptor-gamma coactivator-1alpha; Pink1: PTEN induced putative kinase 1; PTP: permeability transition pore; p38MAPK: p38 map kinase; RCR: respiratory control ratio; Runx1: Runt related transcription factor 1; SDHB: succinate dehydrogenase complex, subunit B; SIRT1: Sirtuin 1; SIRT3: Sirtuin 3; SOL: soleus; SOMMA: Study of Mucle, Mobility, and Aging; SQSTM1: sequestosome-1; TEM: transmission electron microscopy; TIM23: translocase of inner mitochondrial membrane 23; Trk: tyrosine kinase receptors; UnAG: unacylated ghrelin; UQCRC2: ubiquinol-cytochrome c reductase core protein 2; ULK1: Unc-51 like kinase 1; VDAC1: voltage-dependent anion channel; VO2: oxygen consumption; VCO2: carbon dioxide production; Wip2: WPP domain interacting protein 2.

## Discussion

**Figure 4:**
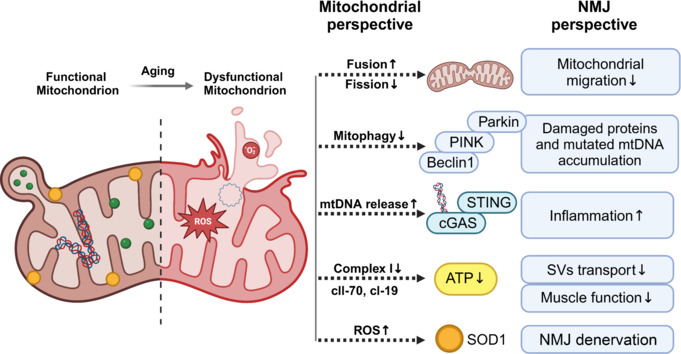
Schematic diagram of the association of mitochondrial dysfunction with NMJ degeneration during aging. Changes in mitochondria during aging are listed on the left, and the effects of these changes on NMJ structure and function are listed on the right. Created with BioRender.com. ATP: Adenosine triphosphate; cGAS: cyclic GMP‒AMP synthase; NMJ: neuromuscular junction; Parkin: Parkin RBR E3 ubiquitin‒protein ligase; PINK: PTEN-induced kinase 1; ROS: reactive oxygen species; SOD1: superoxide dismutase 1; STING: stimulator of interferon genes; SV: synaptic vesicle.

Our systematic review comprehensively examined the alterations in mitochondria within motor units and their profound impact on the structural and functional decline of the NMJ system during aging. The included studies revealed a diverse array of animal species, models, ages, and outcome measures, highlighting the complexity of this research area. A key focus has been placed on mitochondrial quality control, encompassing crucial aspects such as mitochondrial morphology, dynamics of mitochondrial fusion and fission, mitochondrial biogenesis, oxidative stress, and mitophagy during aging. Given the well-established link between mitochondrial dysfunction and redox imbalance in age-related diseases such as sarcopenia, mitochondria have emerged as promising therapeutic targets for preventing neuromuscular aging and its associated pathologies. Furthermore, our review underscores the need for a deeper understanding of the intricate relationships among mitochondrial function, NMJ integrity, and overall muscle health during aging, ultimately informing the development of targeted interventions aimed at preserving muscle function and preventing age-related diseases (**Figure 4**).

The progressive degeneration of motor nerves and NMJs is a critical precursor to the onset of sarcopenia, a condition characterized by muscle fiber atrophy and significant reductions in muscle strength (Bao et al., 2020). Elevated oxidative stress has been shown to impair neurotransmitter release at NMJs in *Sod*^–/–^ mice (Ivannikov and Van Remmen, 2015). Given their high metabolic demands and unique cellular architecture, neuronal cells are particularly vulnerable to mitochondrial dysfunction and subsequent ATP deficits (Klemmensen et al., 2024; Zong et al., 2024). Notably, mitochondria generate approximately 90% of the ATP required for synaptic vesicle (SV) translocation, release, recycling, and refilling at the presynaptic membrane of the NMJ, highlighting their essential role in maintaining normal neuromuscular function (Vos et al., 2010). This intricate relationship between mitochondrial health, neurotransmitter release, and muscle strength underscores the importance of preserving mitochondrial integrity as a key strategy for preventing or delaying sarcopenia.

The accumulation of ROS has been shown to impair Golgi network function, leading to disruptions in synaptic vesicle formation and axonal transport. Consequently, the pool of synaptic vesicles at the NMJ is depleted, resulting in reduced neurotransmitter release (Seager et al., 2020; Duarte et al., 2023). Synaptosome-associated protein 25 (SNAP-25), synaptophysin, and synaptic vesicle proteins are critical for mediating the fusion of synaptic vesicles with the plasma membrane and play essential roles in neurotransmission (Chanaday and Kavalali, 2021). Notably, S-nitrosylation of SNAP-25, which involves the covalent binding of nitric oxide to a cysteine residue, stabilizes the SNAP receptor complex and prevents its disassembly, thereby facilitating efficient vesicle exocytosis (Matsushita et al., 2003; Oh et al., 2024). In contrast, peroxynitrite stimulation leads to an increase in SNAP receptor complex formation and significantly enhances vesicle exocytosis, highlighting the intricate regulation of synaptic transmission by reactive nitrogen species (Bradley and Steinert, 2016).

Mitochondrial swelling, a hallmark of abnormal morphology, is characterized by an influx of water and solutes and serves as a critical morphological feature of cell death (Chen et al., 2023). Mitochondrial permeability transition, triggered by increased intracellular Ca^2+^ and oxidative stress, results in the opening of large conductance permeability transition pores, causing the mitochondrial inner membrane to be permeable to solutes up to 1500 Da into the mitochondrial matrix (Bernardi et al., 2023). This mitochondrial swelling signifies elevated mitochondrial membrane permeability and diminished membrane potential, leading to the release of cytochrome c from the cristae into the cytoplasm (Garcia et al., 2013). In the cytoplasm, cytochrome c interacts with Apaf-1 and dATP/ATP to form an apoptosome that promotes cell death by activating caspase-9 and caspase-3 (Shakeri et al., 2017, 2021). Moreover, mitochondria play pivotal roles in calcium sequestration and release, contributing to calcium homeostasis and providing ATP to fuel Ca^2+^-ATPases, which are critical for SV exocytosis and endocytotic recycling (Li et al., 2025). As individuals age, impairments in the mitochondrial oxidative respiratory chain lead to inadequate ATP production and Ca^2+^ overload, resulting in reduced production and release of SVs at the NMJ (Ivannikov and Van Remmen, 2015; Sataranatarajan et al., 2015). The relationships between the frequency and intensity of synaptic activity, presynaptic mitochondrial quantity, and metabolic function are well established. However, the temporal changes and underlying molecular mechanisms during aging remain poorly defined. Progressive mitochondrial degeneration within presynaptic membranes not only disrupts neurotransmitter release but also results in ATP deficits, increased ROS production, pro-apoptotic protein release, reduced autophagy and imbalanced calcium homeostasis. Collectively, these events can cause neuronal cell death, accelerate NMJ denervation, and exacerbate sarcopenia. Given the critical role of mitochondrial functionality in preserving NMJ integrity and the heightened vulnerability of neuronal mitochondria, targeting these organelles represents a promising therapeutic strategy for the early intervention and management of sarcopenia.

Mitochondrial dynamics are maintained by a regulated cycle of fusion and fission events, with each mitochondrion undergoing approximately 5 fusion‒fission cycles every hour (Twig et al., 2008). This process facilitates intermitochondrial communication, enabling the exchange of proteins, mtDNA, lipids, and metabolites. The regulation of mitochondrial fusion is mediated by proteins such as MFN1, MFN2 and OPA1 (Chen et al., 2023). During energy-deficient states, mitochondria tend to fuse and elongate with increased cristae, increasing ATP synthase activity to maintain ATP output and resist autophagic degradation (Yao et al., 2019). The presence of giant mitochondria at the NMJ axon terminals in aged rats has been observed as a potential protective mechanism (Garcia et al., 2013). However, Ibebunjo et al. (2013) reported conflicting results regarding the expression levels of *Mfn1* and *Mfn2* in rat muscle with age, whereas a study by Ascenzi et al. (2019) reported no such decline in the mRNA levels of *Mfn2* in the muscle of aged WT mice. Furthermore, research in *Drosophila* has shown that overexpression of mitofusin 2/mitochondrial assembly regulatory factor leads to mitochondrial hyperfusion and a decrease in synaptic boutons (Cheon et al., 2023). However, enhanced mitochondrial fusion has been recognized as a self-protective mechanism in various longevity models (Liu et al., 2020), suggesting that the role of mitochondrial fusion in NMJ degeneration during aging and sarcopenia remains unclear. It is essential to further investigate the changes in and roles of MFN1, MFN2, and OPA1 in mitochondrial assembly, migration and mitophagy. Dynamic changes in mitochondrial fusion and division significantly affect mitochondria-to-mitochondria communication and cell-to-cell mitochondria transfer. However, whether this mitochondria-based communication affects Schwann cells, motor neurons, muscle stem cells, satellite cells, and macrophages in motor units is still unclear. This research should be conducted with more standardized animal models and potentially human samples. The aim is to clarify the role of mitochondrial dynamics as a potential target in slowing aging or even extending lifespan (Cheon et al., 2023; Tezze et al., 2023).

Mitophagy, a process that is integral to maintaining mitochondrial function, involves the selective digestion of damaged proteins and mtDNA within mitochondria (Twig and Shirihai, 2011; Thomas and Gustafsson, 2013). Typically, fragmented mitochondria, characterized by reduced size and high levels of *Fis1*, are preferentially targeted for autophagic degradation (Ihenacho et al., 2021). However, it can have far-reaching consequences, including the secretion of mitochondria-derived vesicles into the extracellular space, which may contribute to immune processes, sarcopenia, progeroid conditions, and neurodegenerative diseases such as Parkinson’s disease and Alzheimer’s disease (Picca et al., 2020a, b; Boardman et al., 2023; Zecchini et al., 2023). The observation that mitochondrial quality control disorders at the NMJ presynaptic membrane may precede similar impairments at the postsynaptic membrane and muscle tissues during aging highlights the need for further investigation. Notably, Ibebunjo et al. (2013) reported a significant decrease in the mRNA levels of *Fis1* and *Drp1* in aged rat muscle; however, studies on mitochondrial fission at the NMJ remain scarce. The potential therapeutic targeting of mitochondrial fission is also controversial. Overfragmentation of mitochondria by DRP1 has been implicated in the pathogenesis of neurodegenerative diseases (König et al., 2021). Conversely, reports have suggested that overexpression of DRP1 at mid-life can prolong the lifespan of *Drosophila* (Rana et al., 2017). These contrasting findings underscore the complexity of the interaction between mitochondrial fission and mitophagy, emphasizing the need to consider these factors when designing therapies that target mitochondrial fission and mitophagy.

mtDNA is inherently susceptible to mutations because of its lack of histone protection and proximity to oxidative reactions within mitochondria, despite the presence of repair systems (Wolf, 2021; Glynos et al., 2023). The processes of mitochondrial fusion, fission and mitophagy play crucial roles in maintaining mtDNA integrity. However, as senescence progresses, the mitochondrial quality control system becomes increasingly compromised, affecting these processes (Bazzani et al., 2022). Recent evidence has linked mtDNA to aging and degenerative diseases triggered by chronic inflammation, highlighting its role as a damage-associated molecular pattern (Picca et al., 2018). An accumulation of mtDNA deletion breakpoints and a concomitant reduction in the mtDNA copy number in mouse muscle and neurons with aging have been correlated with cytochrome c oxidase deficiency, a marker of mitochondrial dysfunction (Rygiel et al., 2014; Garcia et al., 2018). Furthermore, in the absence of active caspases, mtDNA has been identified as a trigger for the innate immune cGAS/STING pathway, linking mitochondrial damage to the activation of immune responses (McArthur et al., 2018). cGAS contains a nucleotidyltransferase structural domain and two DNA-binding structural domains. cGAS binds to leaking mtDNA and forms a 2:2 complex that catalyzes the synthesis of cyclic GMP-AMP (cGAMP) from ATP and GTP. cGAMP serves as a second messenger, leading to the activation of STING on the ER, promoting the translocation of nuclear factor-kappa B into the nucleus (Chen et al., 2016). Gulen et al. (2023) reported that mtDNA could activate the cGAS-STING signaling pathway in aged microglia and the central nervous system. Intervention to block the cGAS/STING signaling pathway has shown promise, as it inhibits the inflammatory response while also enhancing cognitive function and muscle strength in aged mice. The p65 subunit of nuclear factor-kappa B directly interacts with and suppresses the activity of PGC-1α (Rabinovich-Nikitin et al., 2022). PGC-1α inhibits the phosphorylation of p65 and consequently reduces its transcriptional activity (Huang et al., 2024). These findings suggest that mtDNA may provoke chronic inflammatory and degenerative diseases through the exchange of mitochondrial-derived vesicles between motor neurons, NMJs, and muscle tissues. Furthermore, elevated levels of mtDNA, both in the circulation and within the extracellular matrix, may serve as biomarkers for chronic inflammation and degenerative diseases. These findings suggest that targeting mtDNA-induced inflammation could be a potential therapeutic approach to mitigate the effects of aging and associated diseases.

Copper-zinc-superoxide dismutase plays a crucial role in mitigating oxidative stress by catalyzing the dismutation of superoxide radicals into oxygen and hydrogen peroxide. Studies using *Sod1*^–/–^ mice are similar with respect to the sarcopenia observed in aging mice in general, such as oxidative stress, NMJ denervation, AChR fragmentation, and muscle atrophy (Jang et al., 2010; Larkin et al., 2011). Muscle-specific deletion of *Sod1* or a neuron-specific reduction in *Sod1* in mice is not sufficient to initiate a full-blown sarcopenia phenotype (Zhang et al., 2013; Sataranatarajan et al., 2015). In contrast to expectations, skeletal muscle-specific *Sod1*^–/–^ mice display unaltered muscle mass, unchanged levels of ROS, preserved mitochondrial ATP production, and an absence of adaptive stress responses in skeletal muscle. The decrease in muscle strength observed in m*Sod1*^–/–^ mice is attributed to NMJ dysfunction, whereas the intrinsic contractile properties of the muscle itself remain intact, as evidenced by normal force generation upon direct stimulation (Zhang et al., 2013). Deterioration of NMJ transmission function occurs prior to structural disintegration of the NMJ and compromised muscle contractility, resulting in an earlier decrease in muscle strength. The expression of copper-zinc-superoxide dismutase specifically in the neuronal tissue of *Sod1*^–/–^ mice conferred protection against muscle atrophy, weakness, and NMJ degeneration. These results suggest that altered redox levels in motor neurons are important factors in the progression of sarcopenia. Furthermore, Fischer et al. (2011) demonstrated that expressing SOD1 exclusively in the mitochondrial intermembrane space could ameliorate the denervated NMJ and motor deficits observed in 12-month-old *Sod*^–/–^ mice (Fischer et al., 2011). These findings underscore the critical role of mitochondrial SOD1 in maintaining the morphological and functional integrity of NMJs. Collectively, these findings provide significant insights into the role of mitochondrial health, particularly the function of SOD1 within the mitochondrial intermembrane space, in preserving NMJ function and structure. Future research should aim to delineate the precise mechanisms through which mitochondrial dysfunction contributes to NMJ degradation and muscle atrophy, potentially revealing new therapeutic targets for age-related neuromuscular decline.

There are several limitations in this article. One major limitation of this study is the heterogeneity among the animal models used, including Albino Swiss mice, C57BL/6 mice, Wister rats, SD rats, and Fischer 344xBrown Norway rats, which introduces variability due to differing genetic backgrounds and age definitions, potentially affecting the consistency of the findings. Furthermore, most studies did not explicitly state in the article that the researchers were blinded to the method of intervention for the animals and that the outcome assessors were blinded. Finally, this review provides little discussion of the role of mtDNA, mitochondrial dynamics, mitochondrial transport, and other mitochondrial functions in the interaction between NMJ degeneration and muscle atrophy.

According to the included articles, there have been a growing number of studies on interventions that target mitochondrial function to improve NMJ degeneration and sarcopenia in recent years. The main interventions include neurotrophic agents, antioxidant drugs, stem cell transplantation, ketogenic diet, and alterations in mitochondrial gene therapy. However, despite the importance of motor neuron degeneration preceding muscle decline and denervation accelerating muscle strength loss, there is a significant gap in research targeting early intervention at the NMJ and motor neurons for sarcopenia during aging. This oversight highlights the need for more targeted research approaches that consider the unique characteristics and dynamics of the NMJ and its interactions with mitochondria.

In conclusion, the aging process and progression of sarcopenia are accompanied by significant alterations in mitochondrial structure, membrane permeability, mitochondrial dynamics, mitophagy, mtDNA integrity, mitochondrial biogenesis and redox status within NMJ and muscle tissues. While the specific role of mitochondrial dynamics in NMJ degeneration remains a topic of debate, emerging evidence suggests that mitochondria-derived vesicles containing mtDNA play a significant role in chronic inflammation and inter-organ crosstalk. Most mitochondrial studies have traditionally focused on neurodegenerative diseases or skeletal muscle, but by exploring mitochondrial changes within the NMJ, we may gain a deeper understanding of the mechanisms of mitochondrial dysfunction and oxidative stress governing the interactions between motor nerves and muscles. These findings could provide new perspectives for aging and sarcopenia research, highlighting the importance of considering the NMJ as a critical site for inter-organ communication and disease progression.

## Data Availability

*Not applicable*.
